# Stress Myocardial Perfusion Imaging in the Emergency Department - New Techniques for Speed and Diagnostic Accuracy

**DOI:** 10.2174/157340312801784916

**Published:** 2012-05

**Authors:** Sheri D Harrison, Mark A Harrison, W Lane Duvall

**Affiliations:** Mount Sinai Medical Center, New York, NY, Mount Sinai Division of Cardiology (Mount Sinai Heart), USA

**Keywords:** SPECT myocardial perfusion imaging, chest pain unit, stress-only protocol, CZT SPECT, radiation exposure.

## Abstract

Emergency room evaluations of patients presenting with chest pain continue to rise, and these evaluations which often include cardiac imaging, are an increasing area of resource utilization in the current health system. Myocardial perfusion imaging from the emergency department remains a vital component of the diagnosis or exclusion of coronary artery disease as the etiology of chest pain. Recent advances in camera technology, and changes to the imaging protocols have allowed MPI to become a more efficient way of providing this diagnostic information. Compared with conventional SPECT, new high-efficiency CZT cameras provide a 3-5 fold increase in photon sensitivity, 1.65-fold improvement in energy resolution and a 1.7-2.5-fold increase in spatial resolution. With stress-only imaging, rest images are eliminated if stress images are normal, as they provide no additional prognostic or diagnostic value and cancelling the rest images would shorten the length of the test which is of particular importance to the ED population. The rapid but accurate triage of patients in an ED CPU is essential to their care, and stress-only imaging and new CZT cameras allow for shorter test time, lower radiation doses and lower costs while demonstrating good clinical outcomes. These changes to nuclear stress testing can allow for faster throughput of patients through the emergency department while providing a safe and efficient evaluation of chest pain.

## INTRODUCTION

Chest pain is one of the most common symptoms leading to emergency department visits. Yearly, approximately 5.5 million patients are evaluated for chest pain that may represent an acute coronary syndrome (ACS). Historically, 2% of patients with an ACS are sent home from the emergency room inappropriately [1]. Over a 10-year period, the percentage of emergency department (ED) visits for chest pain in which a diagnosis of ACS was made decreased 44.9%, from 23.6% in 1999–2000 to 13.0% in 2007–2008 [2]. At the same time, the percentage of chest pain visits that resulted in admission, transfer, or death also declined 17.2% [2]. These figures would suggest that more low-risk patients are being triaged in the ED for chest pain. As overcrowded emergency departments continue to see millions of patients annually with chest pain, it is imperative to have efficient protocols to accurately identify patients with coronary ischemia and rapidly exclude those without. Notably, patients with ACS experience more adverse outcomes at times of the highest waiting room census and patient-hours in the ED, emphasizing the need for rapid throughput [3].

With the large number of ED visits for chest pain, there has been substantial growth in the use of diagnostic imaging as part of the evaluation (up 367.6% from 1999-2008, from 3.4% to 15.9%) [2]. While this utilization increases the amount of time spent in the ED [4], imaging often helps physicians correctly rule out certain conditions, avoiding unnecessary treatment or further testing. Currently, for patients presenting with possible ACS, American Heart Association (AHA) guidelines recommend continued monitoring in an ED, chest pain unit (CPU), or inpatient setting with serial biomarker evaluation. For those who rule out for a myocardial infarction, a provocative stress test (exercise or pharmacological) within 72 hours is recommended as an alternative to inpatient admission [5, 6].

## CLINICAL EVALUATION

Initial admission to a chest pain unit hinges on a physician’s suspicion for myocardial ischemia in a patient lacking high-risk features of an acute coronary syndrome. This leaves a wide variety of low-to-intermediate risk patients being triaged to chest pain units. According to Bayes' theorem, the diagnostic power of exercise stress testing is maximal when the pretest probability of coronary artery disease (CAD) is intermediate (30-70%) based on clinical risk factors. For very low- or very high-risk patients, a positive stress test adds little to the post-test probability of CAD [7]. Thus, the first question that needs to be asked of any patient being evaluated for chest pain is whether the patient needs any additional diagnostic testing beyond clinical evaluation.

A recent analysis reviewed 220 young patients aged 23-40 who were evaluated in an emergency department chest pain unit [8]. All had normal or non-diagnostic ECG’s on presentation, ruled out for myocardial infarction by serial biomarkers and did not have a history of coronary artery disease. Each patient then underwent provocative cardiac testing to identify the presence of myocardial ischemia. Of these, only 6 had positive stress test results, 4 were false-positive tests, so only 2 patients (0.9%) under the age of 40 had true positive stress tests. This analysis suggests that the combination of age younger than 40 years, normal ECG and 2 sets of negative biomarkers at least 6 hours apart identified a patient population at *very *low risk for true-positive stress tests and that cardiac stress testing from the ED added little to the diagnostic evaluation. It would be too general to state that patients under the age of 40 in a chest pain unit never need stress testing as part of their workup, but this analysis highlights the limitations of provocative stress testing in *very* low risk populations. Thus it is important to assess pre-test probability using both clinical assessment and established risk calculators to ensure that patients with a <30% risk are, in most cases, not subjected to testing.

### Exercise Treadmill Testing

For selected patients in the emergency room, exercise treadmill testing (ETT) can provide rapid noninvasive risk stratification. ETT is relatively low cost, readily available, easy to perform and provides proven prognostic information [9]. A Science Advisory of the AHA [10] concluded that a symptom-limited ETT after 8-12 hours of evaluation in low-intermediate risk patients is safe. In certain lower-risk patients, ETT has been performed in those without a full set of cardiac biomarkers, and demonstrated no adverse outcomes at 1-month follow-up [11]. For conservative management of chest pain that could represent unstable angina/non-ST elevation ACS, current guidelines recommend ETT without imaging in patients who can exercise and do not have substantial ECG abnormalities that would inhibit interpretation [5, 12].

Functional capacity is an important variable measured by ETT. Higher exercise capacity, measured in metabolic equivalents (METs), is a powerful predictor of cardiovascular events and survival, regardless of age or gender [13-15]. In a recent analysis, Bourque and colleagues prospectively evaluated the burden of ischemia by nuclear imaging in patients who achieved >85% of the maximum age-predicted heart rate (MAPHR) and an exercise capacity of ≥10 METs (high workload) [16]. Patients with ≥10 METs had more than a 5-fold lower prevalence of reversible ischemia and 2.6-fold fewer fixed perfusion defects than those achieving <7 METs (low workload). The prevalence of significant ischemia at high workload was 17-times lower than patients at a low workload. No patients that achieved >85% MAPHR with a high workload, without ST-segment depression, had significant myocardial ischemia on MPI. 

Therefore, from a prognostic standpoint, since there are excellent clinical outcomes for patients achieving ≥10 METs on a Bruce protocol there is little added information from MPI especially when the ECG response is normal and >85% MAPHR is achieved. Many nuclear cardiology laboratories have subsequently adopted these results into their practice by first applying exercise testing alone while having nuclear tracer agents on “standby”. If a patient achieves ≥85% MAPHR and at least 10 METs without ST-segment depression, the exercise test alone is sufficient. If any parameter is not met, then the tracer is injected according to routine protocol and myocardial perfusion imaging is performed.

## MEDICAL RADIATION EXPOSURE

As important as non-invasive imaging is to the diagnosis of coronary artery disease, it must also be recognized that certain tests expose the patient to radiation. Medical radiation accounts for a large portion of the increased per-capita effective radiation dose to Americans over the last 3 decades, with a large component derived from myocardial perfusion imaging. Estimates for effective doses for the cardiac imaging studies are shown in Table **1**. These estimates were obtained by Chen *et al* using multiple systematic reviews and published sources [17]. The radiation dose for myocardial perfusion imaging can vary significantly based on type of isotope, dual isotope versus single isotope imaging as well as imaging protocols, and the estimate in Table **1** is based on consideration of all these factors. In Chen *et al*’s population based study describing radiation exposure from cardiac imaging, 9.5% of people in a health insurance database had undergone at least one cardiac imaging procedure in a 3-year period. Myocardial perfusion imaging accounted for 74% of the cumulative effective dose. The mean cumulative effective dose from all cardiac imaging was 23.1 mSv, whereas the median dose was 15.6 mSv (range 1.5 to 543.7 mSv). For comparison, the background level of radiation from natural sources in the United States is 3 mSv per year. Given that there is no currently accepted level of radiation exposure that is deemed safe, physicians should strive to achieve doses that are “as low as reasonable achievable” to reduce lifetime risk associated with ionizing radiation exposure [17]. The American Society of Nuclear Cardiology (ASNC) and the Food and Drug Administration have emphasized methods of reducing radiation doses, including ensuring appropriate testing, adjusting stress protocols, limiting radiotracer doses and using new technologies [18, 19].

Among the options available to evaluate for the presence of obstructive coronary artery disease, stress echocardiography does have the advantage of lacking radiation exposure to the patient. In addition, echocardiography can provide pertinent information on cardiac chamber size and valvular function. However, drawbacks of this modality include a reduction in image quality in certain patients because of body habitus or pulmonary disease, and difficult interpretation when resting regional wall motion abnormalities are present. Therefore MPI remains in common use and is widely considered an important modality in the diagnostic evaluation of coronary artery disease and chest pain syndromes. 

## MYOCARDIAL PERFUSION IMAGING

For patients at intermediate risk, single photon emission computed tomography (SPECT) myocardial perfusion imaging, with radioactive tracers such as Thallium-201 (Tl-201) and Technetium-99m (Tc-99m), has been the cornerstone of non-invasive testing for obstructive epicardial coronary disease for decades. A normal MPI provides excellent prognostic information, with a cardiac event rate of <1% at 1 year [20-22]. Diagnostic accuracy is enhanced even more when MPI is integrated with clinical data and exercise treadmill testing [23].

### Stress Testing Protocols

**Fig. (1):**
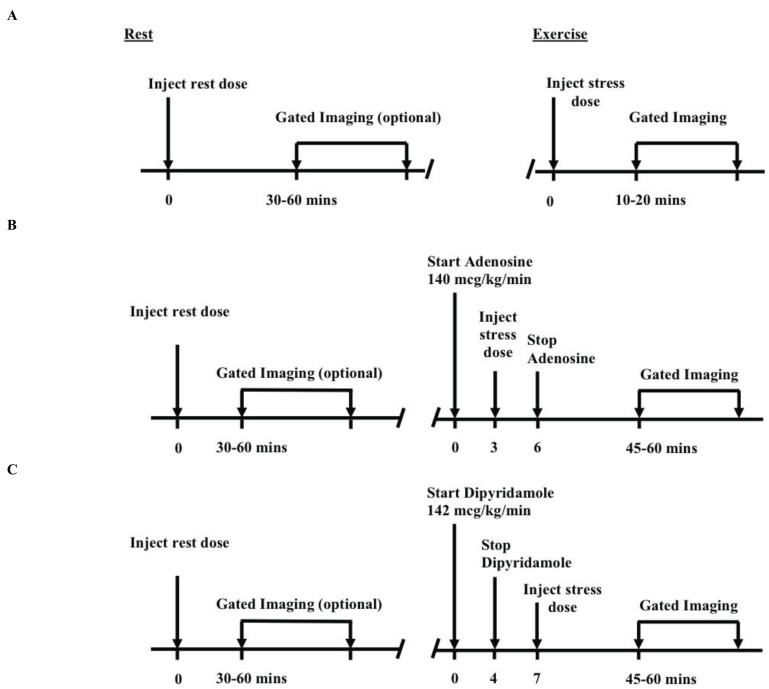
Standard full study (rest-stress) Tc-99m exercise and pharmacologic imaging protocols. **A**. Exercise, **B**. Adenosine, **C**. Dipyridamole [53].

Besides being able to choose between two different isotopes, Tl-201 and Tc-99m, a number of different protocols can be used for a MPI study depending on laboratory preference and patient specific factors including age, gender and body mass index. The low-dose rest followed by high-dose stress sequence has long been the standard protocol of many nuclear laboratories throughout the country (Fig. **1**). For a patient in an emergency-room chest pain unit, a rest-stress sequence takes approximately 3-5 hours to complete [24]. Newer protocols employing low-dose stress first imaging are being adopted by many nuclear laboratories to help address some of the problems with the traditional protocol.

#### Stress-Only Protocols 

**Fig. (2):**
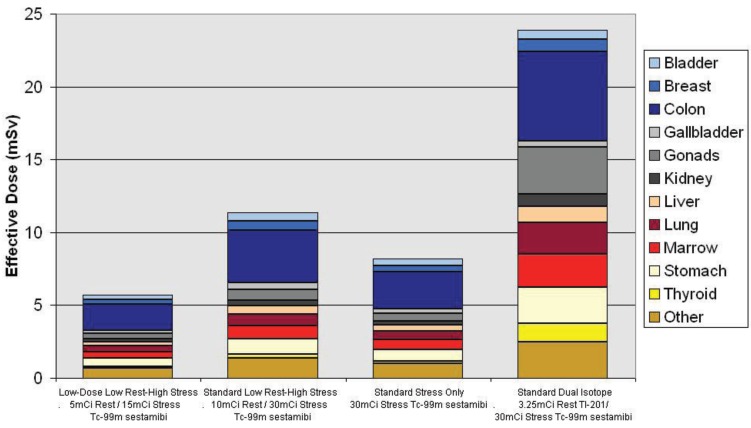
Radiation exposure of various standard and novel stress MPI protocols.

In routine clinical practice, up to 60-70% of appropriately indicated perfusion studies demonstrate normal stress imaging [25-27]. With normal stress images, rest images provide no additional prognostic or diagnostic value and cancelling the rest images would shorten the length of the test which is of particular importance to the ED population. Eliminating the rest-imaging portion allows completion of the entire study in 90 minutes as opposed to the usual 3-5 hours. In addition to a reduction in test length, changing the imaging protocol alone can also lead to significantly lower radiation doses (Fig. **2**). Dual-isotope (Tl-201/Tc-99m) protocols, which can also be shorter, expose patients to significantly more radiation than single isotope testing. Using a stress-only protocol can reduce the radiation doses by approximately 30% in many patients [28].

Despite the potential benefits of stress-only imaging, many laboratories are apprehensive about the reliability, diagnostic accuracy and prognostic ability of a normal stress-only study. The concern often lies in the under-diagnosis of patients with left main or triple vessel CAD that may have a normal appearing stress image [29]. The inclusion of stress symptoms, ECG response, gated images, and attenuation correction can also aid in interpreting a study as normal from stress images only.

In this context, multiple studies have examined the overall clinical utility of stress-only imaging. Gibson *et al* followed 652 patients with low to intermediate probability of CAD who underwent stress-only imaging [29]. After a mean of 22.3 months, the overall cardiac event rate was 0.6% with no cardiac deaths. In a much larger, diverse group of patients, Chang *et al* examined outcomes of 16,854 consecutive patients undergoing stress testing [25]. A stress-only protocol was used in approximately half of the patients evaluated; rest images were obtained only if abnormalities were detected during stress. Over a mean follow-up of 5 years, there was no statistical mortality difference in patients who underwent stress-only imaging or rest/stress imaging. This difference was true regardless of age, sex, clinical risk factors, history of CAD or the stressor used in the test. Similarly, Duvall *et al* examined a retrospective cohort of 10,609 patients who presented for stress testing [28]. Those at lower risk for CAD were assigned to a defined stress-only protocol. Within this group, 1,673 had a normal stress-only study and 3,237 had a normal rest-stress study. At 12 and 40 month follow-up, while controlling for confounding variables, no significant difference was found for both all-cause mortality (p = .94) and cardiac mortality (p = .82).

Another study examined the role of stress-only imaging specifically in an ED CPU setting [30]. A total of 4,145 stress MPIs were performed from the CPU: 2,340 stress-only studies and 1,805 rest-stress. In patients with normal perfusion, at one year of follow-up, there were 11 deaths in the stress-only group (0.5% one-year mortality), and 13 deaths in the rest-stress cohort (1.1% one-year mortality). In addition, the stress-only group had a lower all-cause mortality (p<0.0001) than their rest-stress counterparts. 

The studies reviewed above demonstrate a benign prognosis, similar to that of a full rest-stress study, when stress-only testing is performed in low-risk patients being evaluated for myocardial ischemia. By reducing the time to complete the study, throughput in the emergency room can be significantly affected. In addition to decreasing the overall length of stay, radiation dose to the patient and the cost to the health care system can also be reduced. 

#### Attenuation Correction 

Correct interpretation of images is sometimes challenging due to soft-tissue attenuation and artifacts, which can increase the false-positive rate of the test. Preserved wall motion in the area of a fixed perfusion defect, Q-waves on ECG, as well as overlying soft tissue seen on raw images, can help distinguish artifact from CAD but is by no means fool proof [29, 31, 32]. Attenuation correction using scanning line sources of gadolinium-153 [29, 33] or computed tomography provides a more robust way of discriminating artifact from true perfusion defects than even prone imaging strategies [34]. In a retrospective analysis of 90 patients who underwent stress-only, ECG-gated, Tc-99m sestamibi imaging, Heller *et al.* demonstrated that attenuation correction using gadolinium significantly increased the number of studies that were read as definitely normal/abnormal. The use of attenuation correction led to decreased cost of the study, enhanced laboratory efficiency, shorter study times and lower radiation doses for patients [35].

#### Acute Rest Imaging 

In some institutions, acute rest MPI is used to rapidly risk stratify patients before completing serial assessments of biomarkers. Acute rest MPI has been shown to identify low-and high-risk patients with chest pain [36]. Patients are injected with Tc-99m while they are experiencing symptoms and are imaged when stabilized, providing a snapshot of myocardial perfusion at the time of tracer injection [37]. Perfusion defects indicate ischemia, acute infarction or old infarction and are an independent risk factor for acute myocardial infarction (AMI) [37, 38]. Normal perfusion is associated with a low risk of cardiovascular complications [36-39]. In addition, a review of 11 published articles identified the negative predictive value for acute rest imaging between 99 and 100%. This suggests that patients with normal studies have a very low risk of MI [40]. The value of rest MPI has been demonstrated in multiple trials of ED patients with chest pain, and was shown to lead to significantly lower rates of hospitalization as compared with usual care [39, 41]. A subset analysis in patients with diabetes from a prospective, multicenter, randomized trial demonstrated a reduction in inappropriate hospitalizations when combining acute rest MPI with usual triage decisions, despite the overall higher incidence of coronary disease in diabetic populations [42].

Despite its proven diagnostic accuracy and ability to help risk stratification, acute MPI is not frequently used. Limitations of rest MPI include the inability to distinguish ischemia from an old infarct as recognition of ischemia requires follow-up imaging in a pain-free state to evaluate for resolution of the defect. Also, small areas of ischemia may be missed on MPI alone [9]. Successful implementation of acute rest MPI requires availability of the radiotracers, technologists for injection, imaging equipment and interpreting physicians, thus making coordination difficult after hours in many institutions. In institutions that have developed these protocols, acute rest MPI can help rapidly discharge appropriate patients while reserving further workup for patients with abnormal scans [43].

### Evolving Camera Technology

The original Anger (NaI) gamma camera technology is now over 50 years old and required relatively large amounts of administered radiation, prolonged imaging time, and a large amount of laboratory space. A number of advances have been made in subsequent years including the development of smaller footprint cameras and dual-head cameras which halved the imaging time compared to single head cameras. Iterative reconstruction algorithms with resolution recovery have provided additional reduction in acquisition time with equivalent sensitivity [44]. Further advances, including recently introduced solid-state camera systems have increased sensitivity and resolution by utilizing semiconductors for photon detection. 

#### Cadmium Zinc Telluride Cameras 

The newer solid-state SPECT systems use Cadmium Zinc Telluride (CZT) which can process >10 million photons/second/mm^2^, providing high-energy resolution and very high count rates [44]. The Discovery NM 530c (GE Healthcare, Haifa, Israel) and D-SPECT (Spectrum Dynamics, Caesarea, Israel) high efficiency cameras both employ an array of CZT pixilated detectors and novel collimators based on a multi-pinhole or square-hole design [45]. Compared with conventional SPECT, this CZT camera provides a 3-5 fold increase in photon sensitivity, 1.65-fold improvement in energy resolution and a 1.7-2.5-fold increase in spatial resolution [44]. Studies have demonstrated excellent image quality compared to conventional SPECT using the CZT cameras [46-48], and the newer cameras have excellent diagnostic accuracy in detecting hemodynamically significant coronary artery disease, verified by invasive angiography, with a sensitivity of 94% and specificity of 86%, which is comparable to conventional SPECT imaging [49].

This new technology has allowed for a reduction in imaging time and in administered radiation dose. Image acquisition time has been routinely decreased to 2-4 minutes from 15-20 minutes [46, 47]. Stress first protocols [45], novel dual isotope imaging protocols using a rapid sequential stress Tl-201 and rest Tc-99m imaging protocol of less than 30 minutes in total, as well as simultaneous rest and stress imaging have all been study to decrease overall test time [50, 51]. Radiation exposure reduction using low dose Tc-99m rest-stress protocols with 5 mCi rest and 15 mCi stress dose has also been studied [52]. Image quality, diagnostic performance, and clinical prognosis were maintained even with these low doses and fast acquisition times of 3 and 5 minutes. The effective radiation dose was 5.8 mSv for the rest-stress study, which is almost 49% less than the 11.4 mSv for a conventional 10mCi/30mCi Tc-99m rest-stress study and 76% less than the 23.9 mSv given for a dual isotope study (Fig. **2**) [52].

## CONCLUSION

Emergency room evaluations for the presence of ACS in patients presenting with chest pain continue to rise, though the diagnosis of ACS has declined over recent years. These evaluations, which often include cardiac imaging, are an increasing area of resource utilization in the current health system. Myocardial perfusion imaging from the emergency room remains a vital component of the diagnosis or exclusion of coronary artery disease as the etiology of chest pain and recent advances in camera technology, and changes to the imaging protocols have allowed MPI to become a more efficient way of providing diagnostic information. The rapid but accurate triage of patients in an ED CPU is essential to their care, and stress-only imaging and new CZT cameras allow for shorter test time, lower radiation doses and lower costs while demonstrating good clinical outcomes. These changes to nuclear stress testing can allow for faster throughput of patients through the emergency room while providing a safe and efficient evaluation of chest pain.

## Figures and Tables

**Table 1.** Estimates of Effective Doses for Cardiac Imaging Procedures [17]

Myocardial perfusion imaging study with ejection fraction	15.6 mSv
Cardiac computed tomography for assessment of coronary calcium	3.0 mSv
Cardiac computed tomography with contrast for assessment of coronary arteries	16.0 mSv
Diagnostic coronary angiography	7.0 mSv
Percutaneous coronary intervention	15.0 mSv
